# Contaminated Incubators: Source of a Multispecies Enterobacter Outbreak of Neonatal Sepsis

**DOI:** 10.1128/spectrum.00964-22

**Published:** 2022-06-15

**Authors:** Enrique Hernandez-Alonso, Nadège Bourgeois-Nicolaos, Margaux Lepainteur, Véronique Derouin, Simon Barreault, Adam Waalkes, Luis A. Augusto, Stuti Gera, Orane Gleizes, Pierre Tissieres, Stephen J. Salipante, Daniele de Luca, Florence Doucet-Populaire

**Affiliations:** a Institute of Integrative Biology of the Cell (I2BC), CNRS, CEA, Paris-Saclay University, Gif-sur-Yvette, France; b Department of Bacteriology-Hygiene, AP-HP Paris-Saclay University, Hôpital Antoine Béclère, Clamart, France; c Department of Laboratory Medicine and Pathology, University of Washington, Seattle, Washington, USA; d Department of Neonatal Intensive Care, L’Assistance Publique-Hôpitaux De Paris, Paris-Saclay University, Hôpital Antoine Béclère, Clamart, France; e Physiopathology and Therapeutic Innovation Unit, INSERM U999, Paris-Saclay University, Le Kremlin-Bicêtre, France; Johns Hopkins Hospital

**Keywords:** *Enterobacter*, *Enterobacter bugandensis*, infection control, NICU outbreak, health care-associated infections, sepsis, very low birth weight (VLBW) infants, bloodstream infections

## Abstract

The genus Enterobacter includes species responsible for nosocomial outbreaks in fragile patients, especially in neonatal intensive care units (NICUs). Determining the primary source of infection is critical to outbreak management and patient outcomes. In this investigation, we report the management and control measures implemented during an Enterobacter outbreak of bloodstream infections in premature babies. The study was conducted in a French NICU over a 3-year period (2016 to 2018) and included 20 premature infants with bacteremia. The clinical and microbiological characteristics were identified, and whole-genome sequencing (WGS) was performed on bacteremia isolates. Initially, several outbreak containment strategies were carried out with no success. Next, outbreak investigation pinpointed the neonatal incubators as the primary reservoir and source of contamination in this outbreak. A new sampling methodology during “on” or “in use” conditions enabled its identification, which led to their replacement, thus resulting in the containment of the outbreak. WGS analysis showed a multiclonal outbreak. Some clones were identified in different isolation sources, including patients and neonatal incubators. In addition, microbiological results showed a multispecies outbreak with a high prevalence of Enterobacter bugandensis and Enterobacter xiangfangensis. We conclude that the NICU health care environment represents an important reservoir for Enterobacter transmission and infection. Finally, extracting samples from the neonatal incubator during active use conditions improves the recovery of bacteria from contaminated equipment. This method should be used more frequently to achieve better monitoring of the NICU for HAIs prevention.

**IMPORTANCE** Neonatal incubators in the NICU can be an important reservoir of pathogens responsible for life-threatening outbreaks in neonatal patients. Traditional disinfection with antiseptics is not sufficient to eradicate the microorganisms that can persist for long periods in the different reservoirs. Identification and elimination of the reservoirs are crucial for outbreak prevention and control. In our investigation, using a new strategy of microbiological screening of neonatal incubators, we demonstrated that these were the primary source of contamination. After their replacement, the outbreak was controlled. This new methodology was effective in containing this outbreak and could be a viable alternative for infection prevention and control in outbreak situations involving incubators as a reservoir.

## INTRODUCTION

Health care-associated infections (HAIs) have emerged as a major cause of morbidity, mortality, and rising health care costs within neonatal intensive care units (NICUs) (1). Newborns admitted to the NICU are at high risk of contracting nosocomial infections due to the immaturity of their immune system and the prevalence of invasive procedures (2). One of the most severe HAIs in this context, especially in very low birth weight (VLBW) infants (<1,500 g), is late-onset sepsis (LOS), which is frequently associated with invasive procedures (3–5). HAIs extend hospital stay by 19 days, causing 45.0% of deaths by 2 weeks of age (6). Epidemiological data show that in VLBW infants, the predominant pathogens of neonatal LOS are Gram-positive bacteria (48.0 to 70.0%) such as coagulase-negative staphylococci and Staphylococcus aureus, but Gram-negative organisms (19.0% to 25.0%) such as Enterobacterales are also important (7–9). Over the last decades, the genus Enterobacter has emerged as an important nosocomial pathogen in NICUs (10, 11). Today, more than 20 different species have been identified by molecular techniques (12). Enterobacter spp. can colonize the gastrointestinal tract, as well as surfaces or devices in the NICU, constituting an important reservoir of HAIs (13–15). Improved methodologies for identifying and monitoring outbreaks are necessary to reduce HAIs in NICUs. In this study, we describe management and control measures of a LOS Enterobacter outbreak in a French NICU. We used whole-genome sequencing (WGS) to characterize Enterobacter strain gene content and to provide a comprehensive understanding of the epidemiological dynamics of the outbreak.

## RESULTS

### Outbreak description and demographics.

In May 2016, an outbreak alert was emitted following three cases of Enterobacter sepsis in the NICU. The rate of Enterobacter invasive infections had risen from 0.7% in 2015 to 2.14% in 2016. We initiated an outbreak investigation and surveillance program as follows:

- To exclude cross-transmission, the NICU was divided into two sectors with dedicated health care workers: one with infected and colonized babies and one Enterobacter-free. The movement of neonates within and between units was restricted, and entrance to the outbreak area was kept to a minimum.
- Health care workers’ adherence to the infection control policies (hand hygiene, use of gloves, change of health care clothes and individual protective equipment) was assessed, followed by NICU feedback dissemination, on-site educational and training sessions, and audits of the surveillance measures.
- Biocleaning practices of equipment and hospital environment were audited, and environmental surveillance was introduced.
- Supervision of antibiotic consumption was reinforced.
- The Assistance Publique-Hôpitaux De Paris (AP-HP) infection control team held monthly meetings with the local infection control team (LICT), medical and paramedical NICU infection control staff, and the hospital management to discuss the decision needed to stop the outbreak.
- All parents and visitors were informed of the new hygiene measures and the reason for enhanced infection control of the outbreak and were provided with a written explanation.

**FIG 1:**
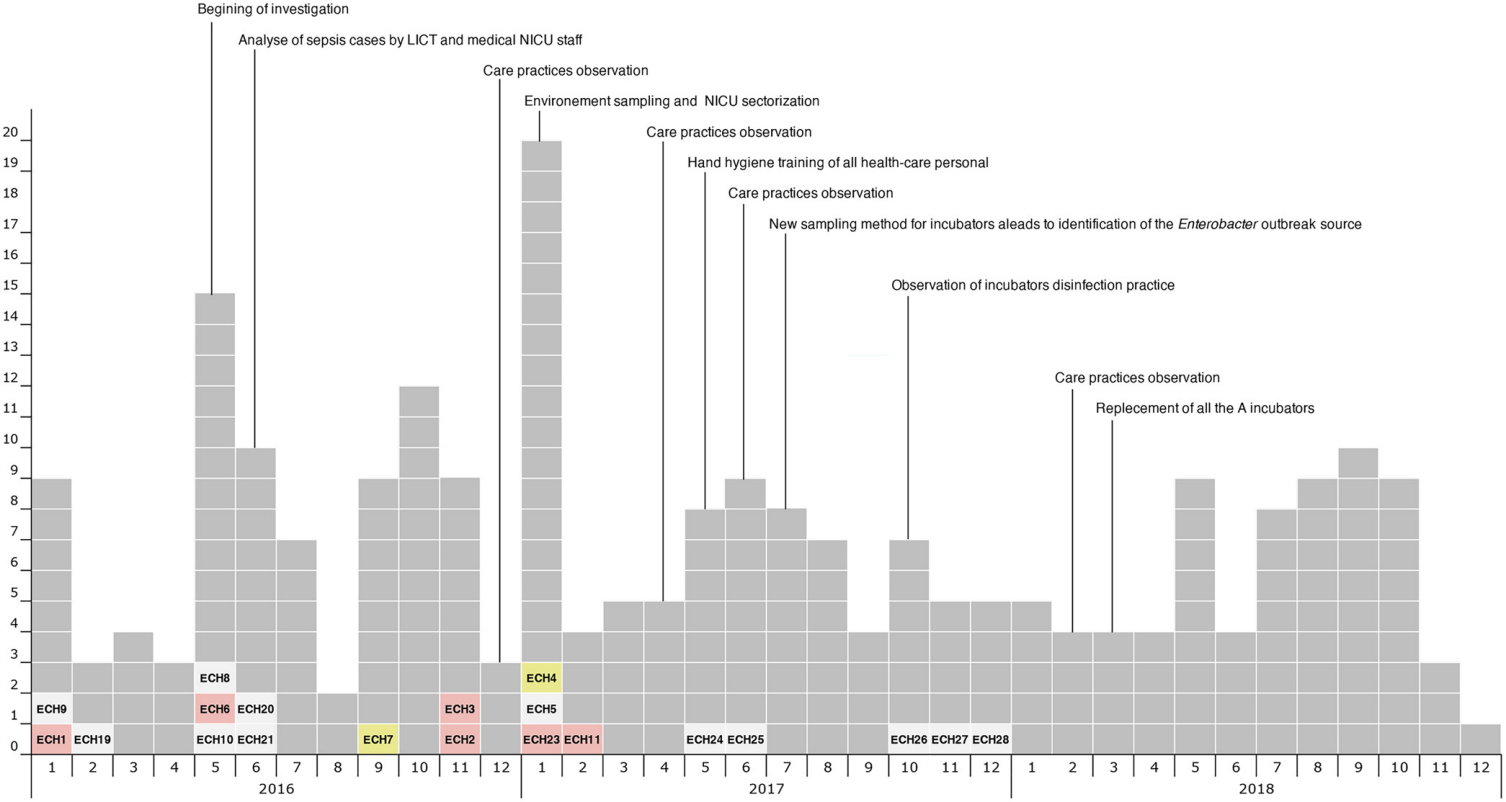
Monthly number of Enterobacter isolates from sepsis and colonized newborns (blood culture/nasopharynx and rectum) from 2016 to 2018 in the neonatal intensive care unit (NICU). Colonization strains are shown in *gray*, and bacteremia strains are identified by ID ECH. *Pink* and *yellow* show lineages A and B, respectively. The figure also shows the timeline of events and the overview of the implementation of the various infection prevention and control measures by the local infection control team. LICT, local infection control team.

Major outbreak control interventions are shown in Fig. 1. From January 2016 to December 2018, 1,621 newborns were admitted to our NICU. During this period, we identified 20 Enterobacter bacteremia cases among 20 separate newborns. In the blood cultures, the 20 strains isolated were all identified as Enterobacter cloacae complex by matrix-assisted laser desorption ionization–time of flight mass spectrometry (MALDI-TOF MS) at the time of the outbreak.

All 20 newborns (100.0%) had a low birth weight (<1,500 g). Mean birth weight was 883.6 ± 343.8 g, gestational age was 27.0 ± 2.1 weeks, 53.0% of the patients were female, 45.0% (9 of 20) of the births were by cesarean, and the mean of CRIB II was 10.0 ± 3.6. Of the 20 patients, 14 (70.0%) died during the outbreak period, and 7 of the 14 patients who died had neutropenia (50.0%).

The highest incidence of bloodstream infection caused by Enterobacter was registered in May to June 2016, November 2016, and January 2017. During these same periods, we identified 220 newborns (13.6%) colonized with Enterobacter. The highest colonization rate was in January 2017 (Fig. 1).

**FIG 2:**
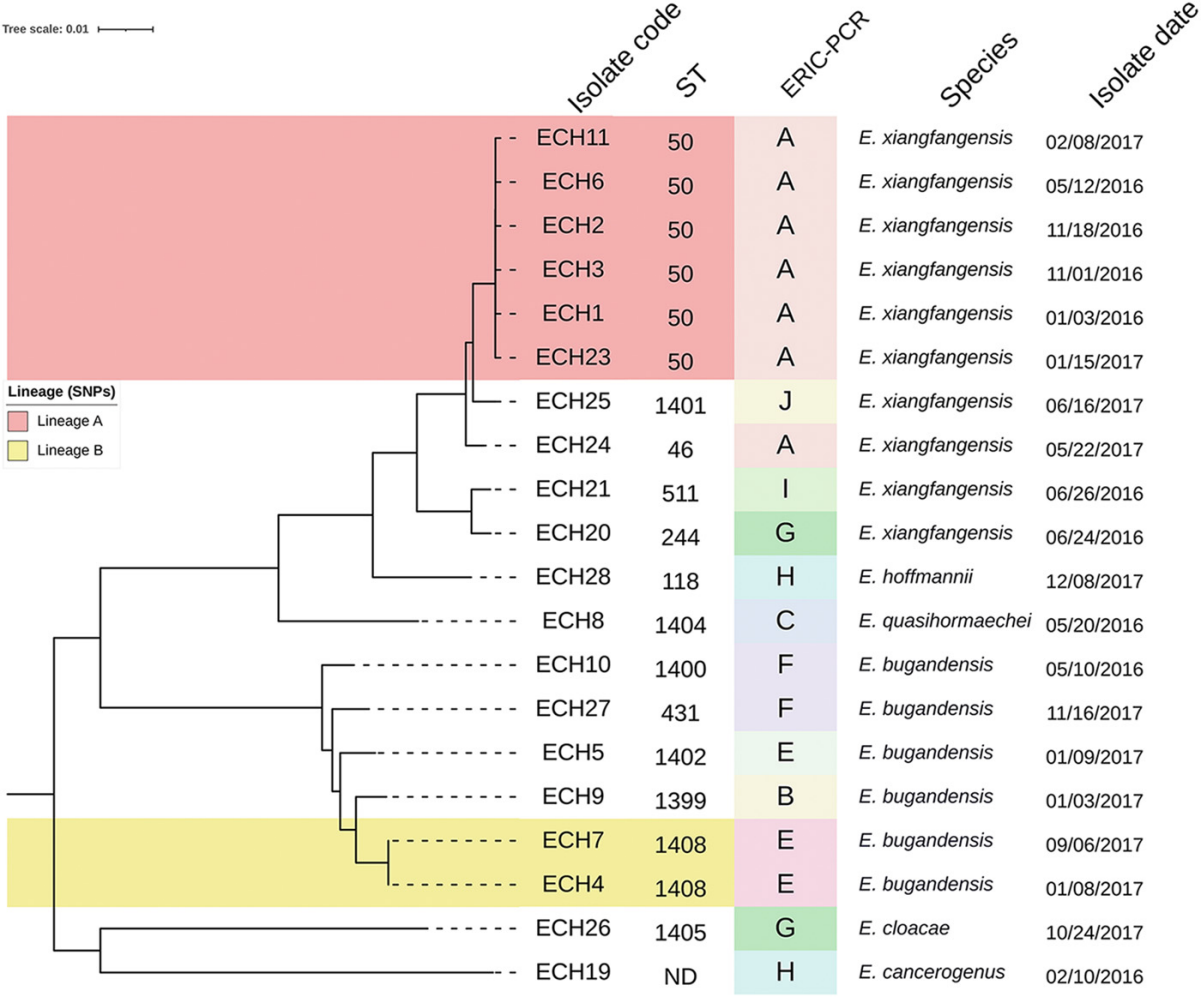
Phylogenetic tree of 20 Enterobacter strains isolated in blood culture during the outbreak period. The phylogenetic tree was performed using the core genome single-nucleotide polymorphism (SNP) analysis by pairwise distance matrix of distinguishing SNPs between the isolates. ERIC, enterobacterial repetitive intergenic consensus; ND, not determined.

The 20 isolates from blood cultures were typed by enterobacterial repetitive intergenic consensus (ERIC)-PCR as the outbreak progressed. It showed 11 different clusters (A-K) (Fig. 2). Two predominant cluster were identified: cluster A (25.0%) and cluster E (23.3%). In addition, cluster A was associated with Enterobacter xiangfangensis, and cluster E was associated with Enterobacter bugandensis.

### Environmental microbiology investigation.

At the start of the outbreak, 100 environmental samples were collected for Enterobacter screening from surfaces, shared devices, water, and drains in the NICU. All the cultures were negative.

**FIG 3:**
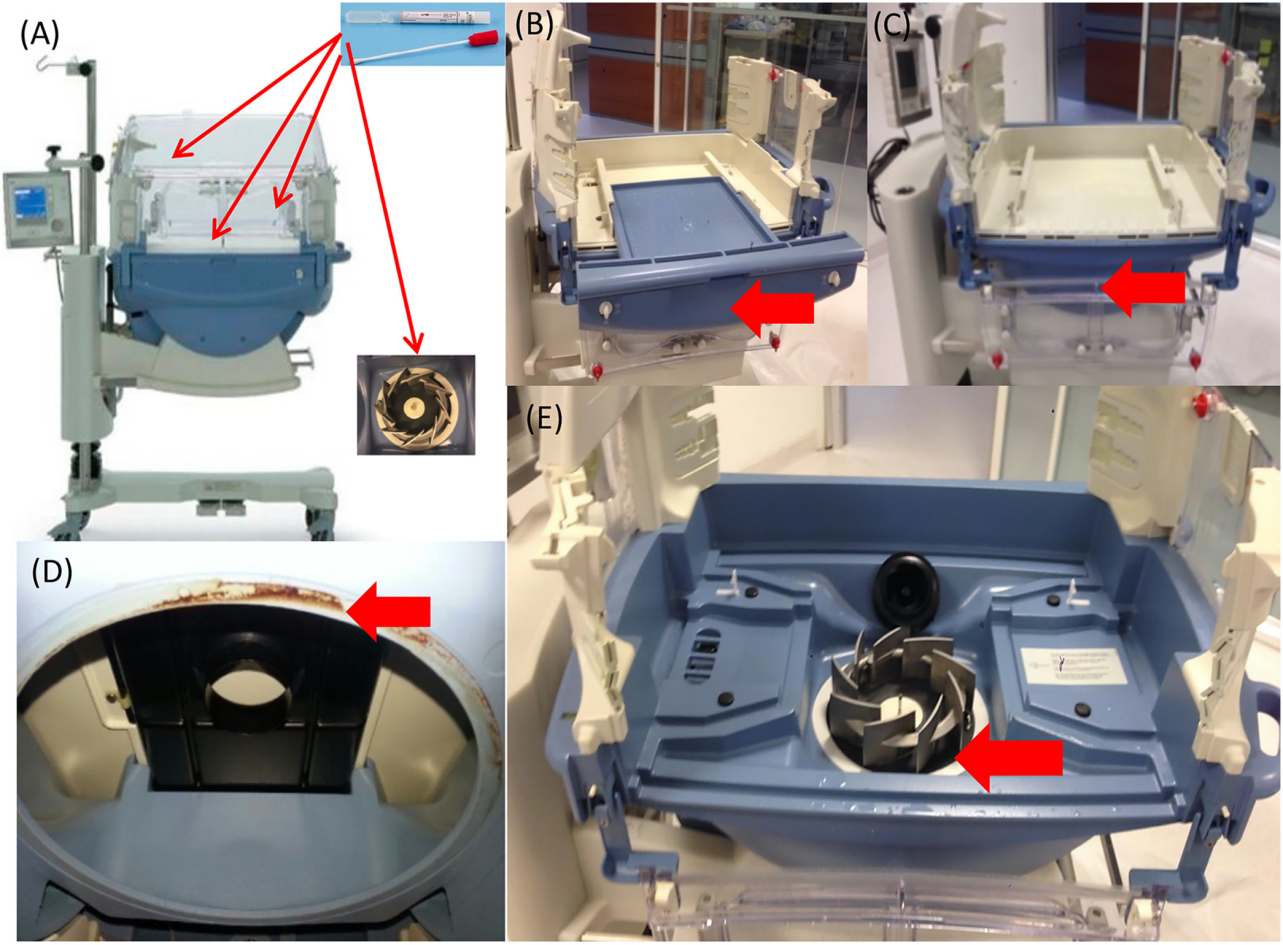
Incubators identified as the source of contamination during the Enterobacter outbreak. (A) Outdoor and indoor sites where microbiological control was performed. (B through E) *Red arrows* show the sites where microbiological controls were performed under “on” conditions that allowed for isolation of the Enterobacter strains.

After the assessment of risk factors, the neonatal incubators seemed to be the most probable source of the outbreak. The local infection control team performed a thorough examination and complete disassembly of the incubators (Fig. 3). The two models of incubators (model A [*n* = 22] and model B [*n* = 11]) were tested in both “off” and active “on” modes between July 2017 and October 2017.

In model A of incubator, 45 samples were collected. Enterobacter was found in 26.0% (5 of 19) in “off” conditions versus 77.0% (20 of 26) in “on” conditions. The results remained positive after changing various motor parts and all seals. In model B, 15 samples were collected. However, Enterobacter was not found in either “off” or “on” conditions.

The 20 Enterobacter strains ECE1-ECE20 isolated from model A incubators were typed by ERIC-PCR. Profiles A, E, F, G, I, and K were identified, showing the same profile clusters as isolates from blood cultures. In addition, the two strains sequenced by WGS showed that ECE1 (profile A) is a member of genetic lineage A, and ECE11 (profile E) constitutes a third lineage C with strain ECH5 (sepsis).

### Genomic analysis of Enterobacter isolates from blood culture.

**FIG 4:**
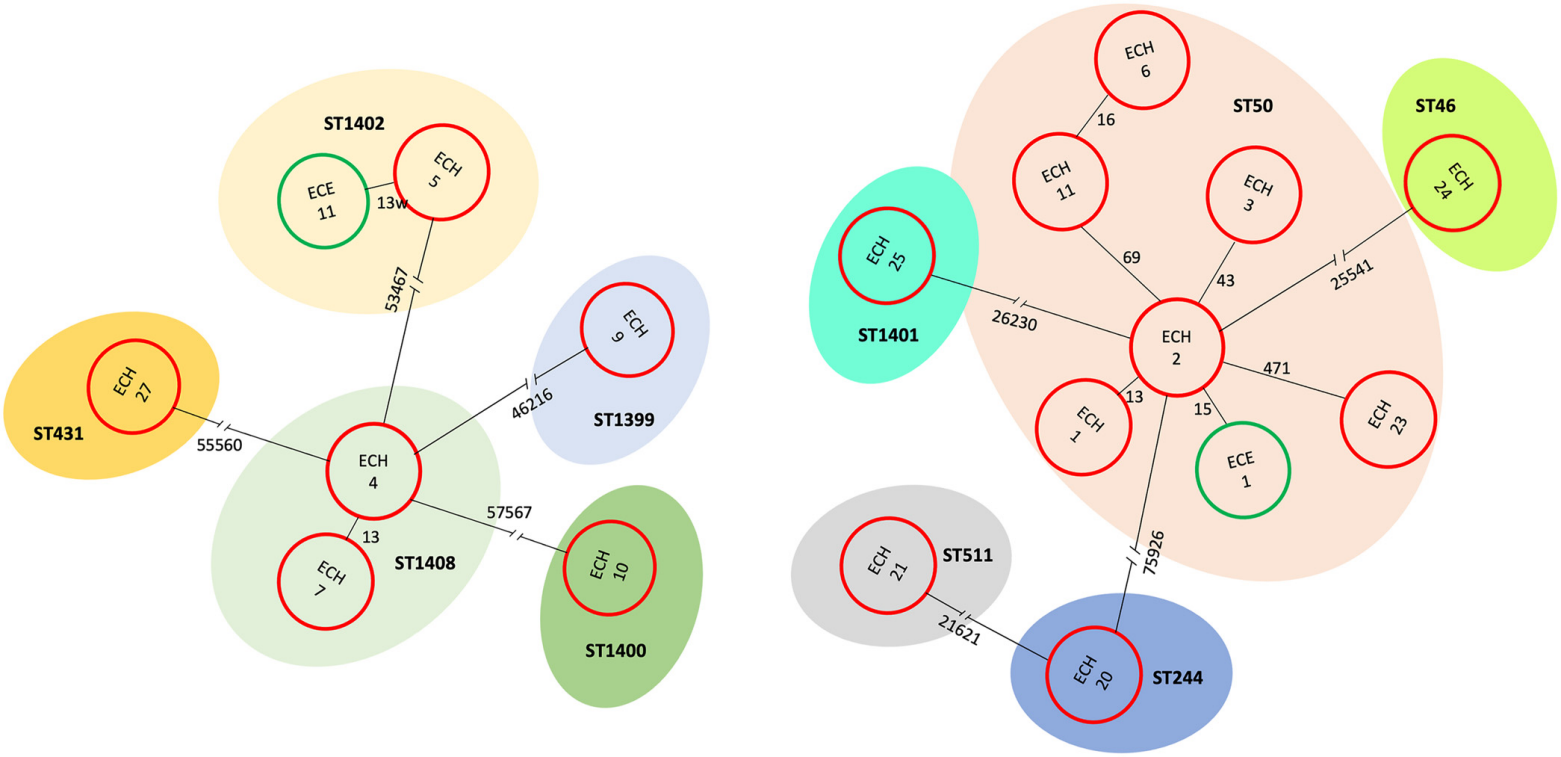
Evolution of pairwise distances by SNP analysis in the strains recovered from bacteremia (*red circles*) and neonatal incubators (*green circles*). (Left) E. bugandensis population. (Right) E. xiangfangensis population. Tree branch numbers indicate SNP distances between genomes (*circles*), and each multilocus sequence type (MLST) is represented with a different color.

We found five species belonging to E. cloacae complex: 50.0% (10 of 20) E. xiangfangensis, 30.0% (6 of 20) E. bugandensis, 5.0% (1 of 20) E. cloacae, 5.0% (1 of 20) Enterobacter hoffmannii, 5.0% (1 of 20) Enterobacter quasihormaechei, and one which did not, 5.0% (1 of 20) Enterobacter cancerogenus. Multilocus sequence type (MLST) analysis distinguished 5 STs among the 10 *E. xiangfangensis* isolates and 5 STs among the 6 *E. bugandensis* isolates, indicating a high genetic diversity (Fig. 2). The ST50 and ST1402 were observed in blood cultures and environmental sources, specifically in neonatal incubators (Fig. 4). In addition, ST50, ST1408, and ST118 persisted during the entire outbreak period (2 years).

A core genome phylogenetic analysis identified two distinct lineages of genetically related isolates (A and B), each of which correlated with different MLST (lineage A, ST50; and lineage B, ST1408) and with ERIC-PCR clusters (lineage A, cluster A; and lineage B, cluster E) (Fig. 2). Lineage A (*n* = 6) isolates were identified as E. xiangfangensis, and lineage B (*n* = 2) isolates were identified as E. bugandensis. Clones were present within each of the lineages, a level of genomic identity that is indicative of direct descent and/or transmission. Within lineage A, ECH1, ECH2, and ECH3 demonstrate five pairwise single-nucleotide polymorphisms (SNPs) and 8 for ECH2 and ECH6. Both members of lineage B were genetically indistinguishable (0 pairwise SNPs) (supplemental file 1).

### Drug susceptibility testing and antibiotic resistance genes.

We determined the antimicrobial and antiseptic susceptibility of the 20 strains recovered from patients with bloodstream infection due to Enterobacter (Table 1). All strains were susceptible to cefepime, aminoglycosides, and ciprofloxacin but resistant to colistin. In addition, in all strains, we observed a heteroresistance to colistin. We found that 30.0% (6 of 20) of the strains were cefotaxime-resistant (CTX-R). All CTX-R strains were identified as E. xiangfangensis, and CTX-R was associated with overproduction of the cephalosporinase AmpC. Lineage-specific patterns of resistance were also observed. Lineage A included 5 of the 6 CTX-R strains. In contrast, lineage B strains were susceptible to CTX. All strains of lineage A carried the AmpC-type β-lactamase ACT-15, even the CTX-S strain ECH23. All lineage A strains showed mutations in AmpR and AmpD. The only differences between the five CTX-R strains and the CTX-S strain (ECH23) were the presence of an insertion (Ser-Ser-Ser-Met) at the amino-terminal end and of a four-amino acid insertion at the carboxyl-terminal end in the AmpD protein in the CTX-R strains. These differences might be associated with the AmpC overproduction (supplemental file 2). The sixth CTX-R strain (ECH24) harbored an ACT-17.

**TABLE 1:** Antimicrobial and antiseptic susceptibility (MICs) of the 20 Enterobacter sepsis strains from neonatal patients in the neonatal intensive care unit^*a*^

Isolate code	Bacteria	ST	ERIC-PCR	Lineage	MIC (mg/liter)																			
					CTX		FEP		MEM		PIP/TZ		CIP		GEN		KN		COL		BZK		CHX	
ECH11	E. xiangfangensis	50	A	A	128	R	1	S	0.06	S	32	R	0.031	S	0.5	S	2	S	128	R	64	DS	>128	DS
ECH6	E. xiangfangensis	50	A	A	16	R	0.5	S	0.06	S	128	R	0.007	S	0.5	S	2	S	16	R	128	DS	>128	DS
ECH2	E. xiangfangensis	50	A	A	64	R	0.25	S	0.06	S	8	S	0.031	S	0.5	S	1	S	32	R	64	DS	128	DS
ECH3	E. xiangfangensis	50	A	A	64	R	0.5	S	0.03	S	8	S	0.007	S	0.5	S	2	S	16	R	128	DS	>128	DS
ECH1	E. xiangfangensis	50	A	A	64	R	0.5	S	0.12	S	64	R	0.003	S	0.5	S	4	S	32	R	64	DS	128	DS
ECH23	E. xiangfangensis	50	A	A	0.25	S	0.03	S	0.03	S	1	S	0.003	S	0.5	S	2	S	8	R	64	DS	>128	DS
ECH25	E. xiangfangensis	1401	J		0.25	S	0.03	S	0.06	S	2	S	0.031	S	0.5	S	2	S	16	R	64	DS	>128	DS
ECH24	E. xiangfangensis	46	A		32	R	0.5	S	0.06	S	4	S	0.031	S	0.25	S	0.5	S	4	R	64	DS	>128	DS
ECH21	E. xiangfangensis	511	I		0.25	S	0.06	S	0.03	S	1	S	0.003	S	0.5	S	2	S	16	R	64	DS	128	DS
ECH20	E. xiangfangensis	244	G		0.25	S	0.06	S	0.03	S	1	S	0.003	S	0.5	S	2	S	8	R	64	DS	>128	DS
ECH28	E. hoffmannii	118	H		0.06	S	0.01	S	0.06	S	4	S	0.125	S	0.5	S	32	R	128	R	64	DS	>128	DS
ECH8	E. quasihormaechei	1404	C		1	S	0.5	S	0.01	S	2	S	0.003	S	0.5	S	2	S	16	R	64	DS	>128	DS
ECH10	E. bugandensis	1400	F		0.5	S	0,06	S	0.06	S	8	S	0.007	S	0.5	S	2	S	128	R	128	DS	>128	DS
ECH27	E. bugandensis	431	F		0.25	S	0.03	S	0.06	S	1	S	0.031	S	0.5	S	2	S	128	R	64	DS	128	DS
ECH5	E. bugandensis	1402	E		0.5	S	0.03	S	0.06	S	1	S	0.015	S	0.5	S	4	S	128	R	128	DS	128	DS
ECH9	E. bugandensis	1399	B		0.5	S	0.06	S	0.03	S	2	S	0.031	S	0.5	S	4	S	128	R	128	DS	>128	DS
ECH7	E. bugandensis	1408	E	B	0.5	S	0.06	S	0.06	S	2	S	0.007	S	1	S	2	S	64	R	64	DS	>128	DS
ECH4	E. bugandensis	1408	E	B	0.5	S	0.06	S	0.06	S	2	S	0.015	S	0.5	S	4	S	64	R	128	DS	128	DS
ECH26	E. cloacae	1405	G		0.5	S	0.5	S	0.06	S	2	S	0.062	S	0.5	S	4	S	16	R	64	DS	>128	DS
ECH19	E. cancerogenus	ND	H		0.5	S	0.06	S	0.06	S	2	S	0.007	S	0.5	S	2	S	32	R	64	DS	>128	DS
ATCC 13047	E. cloacae				0.06	S	0,03	S	0.03	S	4	S	0.031	S	0.5	S	2	S	128	R	32	S	2	S

a ERIC, enterobacterial repetitive intergenic consensus; ST, sequence type; CTX, cefotaxime; FEP, cefepime; MEM, meropenem; PIP/TZ, piperacillin-tazobactam; CIP, ciprofloxacin; GEN, gentamicin; KN, kanamycin; COL, colistin; BZK, benzalkonium chloride; CHX, chlorhexidine; S, susceptible; R, resistant; DS, decreased susceptibility; ND, not determined.

WGS analysis did not show acquisition of resistance-associated genes. Patients were treated with cefepime or piperacillin-tazobactam or meropenem plus gentamicin or amikacin or ciprofloxacin according to the susceptibility of the strain. Finally, decreased susceptibility to the antiseptics evaluated (chlorhexidine and benzalkonium chloride) was observed in all the strains (100%).

## DISCUSSION

In this investigation, we describe the clinical, microbiological and molecular characteristics, as well as the management and control measures, of an Enterobacter outbreak in one NICU over a 2-year time span. Identifying the primary source of infection is critical in the management of an outbreak and of each patient with bacteremia (16). Here, we determined that the incubators were the primary source of Enterobacter strains responsible for the outbreak.

Clinical characteristics of the patients were consistent with the findings of other studies (11, 17). A higher mortality rate (70.0%) was observed in our study compared to other outbreaks of Enterobacter infection in NICUs with reported mortality rates of 34.0 to 63.6% (17–19). Recently, our group highlighted the association of fatal septic shock and the presence of lipopolysaccharide (LPS) modifications that could explain the mortality rate observed in Enterobacter outbreaks (20). The impact of this LPS modification on virulence has also been evidenced in other species such as Salmonella spp. and Acinetobacter spp. (21, 22).

It is known that Enterobacter spp. colonizes the newborn immediately after birth (23, 24). Interestingly, the cases of infection covered by this study did not necessarily occur during periods of high incidence of colonization in the NICU. Furthermore, colonization persisted after even after biocleaning, as has been reported in other studies (11, 13). Enterobacter colonization in newborns follows different patterns of colonization due to limited maternal contact, delayed enteral feeding, antibiotic treatment, and exposure to the NICU environment. The hypothesis that Enterobacter infections classically occur after intestinal colonization and translocation remains moot (11). In our study, gut colonization never preceded sepsis.

The NICU environment plays an important role as a reservoir for invasive strains causing neonatal sepsis (25). The multiclonal nature of our Enterobacter outbreak, quickly elucidated by ERIC-PCR and then by SNPs analysis, supports the hypothesis that cross-contamination in the NICU environment can be a cause of HAIs (25, 26). Transmission of invasive strains usually occurs from patient to patient through the hands of health care workers and through shared devices (27, 28). In this context, premature newborns are especially susceptible to Enterobacter infection due to their immature immune system, their low birth weight, and the invasive procedures they undergo (28, 29).

To control the outbreak, the NICU was divided into two sections in January 2017 to prevent transmission. However, the incidence of sepsis cases, as well as colonization, continued. The presence of Enterobacter and other pathogens in neonatal incubators is common and was suspected to be the source of contamination for HAIs in this NICU (30). We initially screened for Enterobacter spp. in the incubators following traditional procedures, but no Enterobacter strain was isolated (31). In response, the hospital’s bacteriology team decided to carry out a new strategy. They collected specimens with the incubators running, which facilitated bacterial recovery by raising humidity and temperature to more optimal conditions for microorganism growth. Using this new strategy, it was possible to find Enterobacter isolates. In addition, several MLSTs were isolated from multiple sources (blood cultures from patients with bacteremia and incubators), supporting the hypothesis that incubators were the principal source of contamination within the NICU during this outbreak (Fig. 4), as was reported in other works (32, 33). In March 2018, due to the persistence of the outbreak despite the reinforcement of control measures, all 22 model A incubators, contaminated by Enterobacter were replaced. A significant decrease in the number of cases of bloodstream infections due to Enterobacter was observed.

Selective pressures from antimicrobials are another important factor in the emergence of Enterobacter in the hospital environment. Interestingly, in contrast to other studies (29, 34), multidrug-resistant (MDR) strains were not identified in our cohort. Nevertheless, given the link between antimicrobial regimens and colonization of newborns with MDR Enterobacter strains, control of antimicrobial therapy during and after an outbreak should be undertaken to avoid the emergence of potential MDR strains (32). Measures such as revision of antimicrobial therapy and additional training of the NICU staff to reduce antimicrobial consumption and to prevent cross-contamination in the NICU were accordingly implemented by our hospital system. In addition, the prevalence of 100% of decreased susceptibility to quaternary ammonium compounds observed in this study suggests that another method of incubator disinfection such as steam decontamination should be used to reduce the presence of pathogens in the NICU (33).

The prevalence and distribution of specific Enterobacter species in the NICU are not well documented due to frequent misidentification of this pathogen in clinical practice. In several studies where MALDI-TOF MS was implemented as a tool for bacterial identification, Enterobacter was reported as E. cloacae or an E. cloacae complex (13, 26, 28). In our investigation, WGS was used to establish the precise taxonomy of bacterial isolates, revealing E. xiangfangensis and E. bugandensis to be the most prevalent species in the outbreak. E. bugandensis, a recently described species, was first identified as responsible for an NICU outbreak in 2016 (19, 34). In 2018, Pati et al. (35) reported the potential of E. bugandensis for causing bloodstream infections, as well as its ability to induce the release of proinflammatory cytokines. These results support the hypothesis that E. bugandensis is an emerging pathogen in the NICU with a virulence potentially greater than other species of the genus Enterobacter (35, 36). However, more studies implementing tools for precise species identification are needed in additional settings to better understand its epidemiology in the NICU (12).

Although illuminating, our study has some limitations. First and foremost, our study was conducted in a single medical center, which does not authorize us to generalize about the epidemiological dynamics of Enterobacter in all NICUs.

Our study shows the importance of long-term broad surveillance of NICUs to identify the epidemiology of neonatal outbreaks due to the different Enterobacter species and shows the usefulness of WGS in understanding the transmission and prevention of hospital-acquired bloodstream infections. Additionally, we find that sampling neonatal incubators while they are in active use improves recovery of organisms from contaminated instruments. These methods should be employed more generally to achieve better surveillance of the NICUs for HAIs prevention.

## MATERIALS AND METHODS

### Hospital characteristics, patient population, and data collection.

AP-HP is a public health institution administering 38 teaching hospitals spread throughout Paris, its suburbs, and surrounding counties, with 21,000 beds (10% of all public hospital beds in France). It serves 12 million inhabitants. Antoine-Béclère Hospital is a 400-bed teaching AP-HP hospital providing primary care to adults and neonatal patients, including a level 3 NICU with 28 intensive care beds. A local infection control team (LICT) oversees the prevention and surveillance of HAIs in the hospital. Clinical data at birth included gestational age, weight, cesarean birth, Clinical Risk Index for Babies (CRIB) scoring and postnatal neutropenia (<1,000 polynuclear neutrophils/μL). HAIs were defined by positive blood culture ≥ 48 h from NICU admission.

### Outbreak management.

In May 2016, a significant rise in bacteremia due to Enterobacter spp. was observed in the NICU. According to the accepted definition, an outbreak due to Enterobacter was suspected (37). Surveillance cultures of rectal and cavum swabs were obtained from all admissions to the NICU. Colonization was defined as a rectal/cavum swab sample that tested positive for Enterobacter. Soon after the confirmation of the outbreak, neonatal HAI prevention actions were implemented by the LICT.

### Environment investigation.

In January 2017, the LICT set up an environment sampling campaign to identify a possible environmental source of the outbreak. Multiple environmental sites were tested, including shared devices in the ward (gloves, sheets, plaster, ultrasound gel, neonatal incubators, etc.) using contact plates or swabs. In addition, water samples were collected in different rooms and filtered to search for Enterobacter spp., and multiple siphons were swabbed. The swabs were inoculated on Columbia agar with 5.0% sheep blood and Drigalski agar plates (bioMérieux SA, Marcy l’Etoile, France). The isolated colonies were identified using the reference spectra library of the Bruker Biotyper MALDI-TOF MS (Bruker Daltonics). After the absence of identification of the contamination source and as the outbreak was still active, in July 2017, we implemented two different incubator sampling protocols. Both methodologies included the sampling of the corners and of risky and unattainable areas (seals, ventilator, holes, etc.) just after cleaning. The first method was performed under “off” conditions, and the second was performed under “on” conditions, which were 37°C, and 85.0% humidity for 48 h. Both methods were used on the two incubator models (model A, *n* = 22; and model B, *n* = 11) owned by the NICU.

### Microbiology diagnostic.

Blood cultures were processed for the diagnosis of bacteremia with automated microbial detection systems BacT/Alert 3D system (bioMérieux SA, Marcy l’Etoile, France). To determine the evolution of HAIs in neonatal patients and colonized babies, all newborns admitted were routinely screened for bacterial colonization and received a nasopharynx and rectum examination on their arrival in the unit and on a weekly basis following the admission. Rectal and cavum swabs collected from patients and surfaces were inoculated on Drigalski agar (bioMérieux SA, Marcy l’Etoile, France). All inoculated samples were incubated at 36°C for 48 h. The isolates recovered were routinely identified using MALDI-TOF MS.

### Antimicrobial and antiseptic susceptibility evaluation.

MICs of cefotaxime (CTX), cefepime (FEP), meropenem (MEM), piperacillin-tazobactam (PIP/TZ), ciprofloxacin (CIP), gentamicin (GEN), kanamycin (KN), and colistin (COL) were determined by the Mueller–Hinton broth microdilution method. Interpretation followed the recommendations of the European Committee on Antibiotic Susceptibility Testing (EUCAST) (38). MICs of chlorhexidine (CHX) and benzalkonium chloride (BZK) were determined by Mueller–Hinton broth microdilution method in accordance with Clinical and Laboratory Standards Institute guidelines (CLSI, 2019). Antiseptic decrease susceptibility was acknowledged if the MIC was less or equal to 2 μg/mL in keeping with previous reports (39, 40). Each antimicrobial and antiseptic susceptibility determination was performed three times. Escherichia coli ATCC 2592 and E. cloacae ATCC 13047 were used as quality control in each run.

### Strains molecular typing by ERIC-PCR.

To quickly identify the clonal relatedness of Enterobacter strains during the outbreak period, an ERIC-PCR was designed. DNA extraction was performed with the Easy Mag kit (bioMérieux, France), and 2 μL was used as the DNA templates. Subsequently, the amplification was performed using ERIC2 primers: 5′-AAGTAAGTGACTGGGGTGAGCG-3′. The amplification reaction volume was 25 μL under the following conditions: an initial denaturation for 10 min at 94°C, followed by 40 cycles with amplification at 94°C for 30 s, 55°C for 30 s for alignment, elongation stage at 72°C for 1 min, and a final stage of 10 min at 72°C. The amplified products were resolved through electrophoresis and analyzed on 1.5% agarose with GelRed revelator (Biotium, USA). Patterns of different strains were compared by visual inspection, as described by Coudron et al. (41). The patterns were interpreted as identical if an identical number of bands of the same size was found.

### Whole-genome sequencing and analysis.

Sequencing libraries were prepared using the Nextera XT DNA sample preparation kit (Illumina, San Diego, CA, USA) according to the manufacturer’s instructions. We multiplexed and sequenced samples on an Illumina NextSeq500. We obtained *de novo* assembly using SPAdes assembler version 3. 10. 1. The bacterial genome was annotated using the Rapid Annotation Subsystem Technology (RAST) online server. Antibiotic resistance genes were further investigated using the Resistance Gene Identifier (RGI) of the Comprehensive Antibiotic Resistance Database (CARD) and ResFinder (https://cge.cbs.dtu.dk/services/ResFinder/). Assignment of isolates to species was ascertained by BLAST and ANIB analysis using pyANI (42). The core genome was determined as 1,106 genes. For the investigation of molecular epidemiology, a core genome SNP analysis was performed. Reads were trimmed using fastq-mfc from ea-utils-1.1.2.779 (43), and *de novo* genome assembly of isolates was performed using ABySS 2.0.2 (44). A core genome alignment was created from these assemblies using recombination-adjusted method (roary version 3.13.0) (45) with -s and -e flags. FastTree v2.1.8 was then used to construct a phylogenomic tree (46). snp-dists v0.8.2 (47) was used to construct a pairwise distance matrix for distinguishing SNPS between the isolates. Using thresholds previously established for Enterobacterales, we defined clonality as ≤10 pairwise SNPs in the core genome (48).

### Ethics approval.

The study was approved by the ethical committee of the French Society of Intensive Care (CE SRLF 19–40).

### Data availability.

The genome sequencing data are publicly available at the NCBI GenBank under BioProject accession number PRJNA770343.
