# Supplementary material for: Contaminated Incubators: Source of a Multispecies Enterobacter Outbreak of Neonatal Sepsis
Source: Microbiol Spectr. 2022 Jun 15;10(4):e00964-22. doi: 10.1128/spectrum.00964-22 (PMC9430301; doi:10.1128/spectrum.00964-22)
Supplement: Supplemental file 2 — Supplemental material. Download spectrum.00964-22-s0002.pdf, PDF file, 0.1 MB [file spectrum.00964-22-s0002.pdf]

AmpR alignement

```
ECH1                  MTRSYLPLNSLRAFEAAARHLSFTHAAIELNVTHSAISQHVKTLEQHLNCQLFVRVSRGL 60
ECH2                  MTRSYLPLNSLRAFEAAARHLSFTHAAIELNVTHSAISQHVKTLEQHLNCQLFVRVSRGL 60
ECH3                  MTRSYLPLNSLRAFEAAARHLSFTHAAIELNVTHSAISQHVKTLEQHLNCQLFVRVSRGL 60
ECH6                  MTRSYLPLNSLRAFEAAARHLSFTHAAIELNVTHSAISQHVKTLEQHLNCQLFVRVSRGL 60
ECH11                 MTRSYLPLNSLRAFEAAARHLSFTHAAIELNVTHSAISQHVKTLEQHLNCQLFVRVSRGL 60
ECH24                 MTRSYLPLNSLRAFEAAARHLSFTHAAIELNVTHSAISQHVKTLEQHLNCQLFVRVSRGL 60
ECH23           CTX-S MTRSYLPLNSLRAFEAAARHLSFTHAAIELNVTHSAISQHVKTLEQHLNCQLFVRVSRGL 60
ATCC13047             MTRSYLPLNSLRAFEAAARHLSFTHAAIELNVTHSAISQHVKALEQHLNCQLFVRVSRGL 60
                      ******************************************:*****************

ECH1                  MLTTEGENLLPVLNDSFDRIAGMLDRFANHRAQEKLKIGVVGTFATGVLFSQLEDFRRGY 120
ECH2                  MLTTEGENLLPVLNDSFDRIAGMLDRFANHRAQEKLKIGVVGTFATGVLFSQLEDFRRGY 120
ECH3                  MLTTEGENLLPVLNDSFDRIAGMLDRFANHRAQEKLKIGVVGTFATGVLFSQLEDFRRGY 120
ECH6                  MLTTEGENLLPVLNDSFDRIAGMLDRFANHRAQEKLKIGVVGTFATGVLFSQLEDFRRGY 120
ECH11                 MLTTEGENLLPVLNDSFDRIAGMLDRFANHRAQEKLKIGVVGTFATGVLFSQLEDFRRGY 120
ECH24                 MLTTEGENLLPVLNDSFDRIAGMLDRFANHRAQEKLKIGVVGTFATGVLFSQLEDFRRGY 120
ECH23           CTX-S MLTTEGENLLPVLNDSFDRIAGMLDRFANHRAQEKLKIGVVGTFATGVLFSQLEDFRRGY 120
ATCC13047             MLTTEGENLLPVLNDSFDRIAGMLDRFANHRAQEKLKIGVVGTFATGVLFSQLDDFRRSY 120
                      *****************************************************:****.*

ECH1                  PHIDLQLSTHNNRVDPAAEGLDYTIRYGGGAWHGTEAEFLCHAPLAPLCTPDIAASLHSP 180
ECH2                  PHIDLQLSTHNNRVDPAAEGLDYTIRYGGGAWHGTEAEFLCHAPLAPLCTPDIAASLHSP 180
ECH3                  PHIDLQLSTHNNRVDPAAEGLDYTIRYGGGAWHGTEAEFLCHAPLAPLCTPDIAASLHSP 180
ECH6                  PHIDLQLSTHNNRVDPAAEGLDYTIRYGGGAWHGTEAEFLCHAPLAPLCTPDIAASLHSP 180
ECH11                 PHIDLQLSTHNNRVDPAAEGLDYTIRYGGGAWHGTEAEFLCHAPLAPLCTPDIAASLHSP 180
ECH24                 PHIDLQLSTHNNRVDPAAEGLDYTIRYGGGAWHGTEAEFLCHAPLAPLCTPDIAASLHSP 180
ECH23           CTX-S PHIDLQLSTHNNRVDPAAEGLDYTIRYGGGAWHGTEAEFLCHAPLAPLCTPDIAASLHSP 180
ATCC13047             PHIDLQLSTHNNRVDPAAEGLDYTIRYGGGAWHGTEATFLCSAPLAPLCTPDIAAALHTP 180
                      ************************************* *** *************:**:*

ECH1                  ADILRFTLLRSYRRDEWTAWMQAAGEHPPSPTHRVMVFDSSVTMLEAAQAGVGIAIAPVD 240
ECH2                  ADILRFTLLRSYRRDEWTAWMQAAGEHPPSPTHRVMVFDSSVTMLEAAQAGVGIAIAPVD 240
ECH3                  ADILRFTLLRSYRRDEWTAWMQAAGEHPPSPTHRVMVFDSSVTMLEAAQAGVGIAIAPVD 240
ECH6                  ADILRFTLLRSYRRDEWTAWMQAAGEHPPSPTHRVMVFDSSVTMLEAAQAGVGIAIAPVD 240
ECH11                 ADILRFTLLRSYRRDEWTAWMQAAGEHPPSPTHRVMVFDSSVTMLEAAQAGVGIAIAPVD 240
ECH24                 ADILRFTLLRSYRRDEWTAWMQAAGEHPPSPTHRVMVFDSSVTMLEAAQAGVGIAIAPVD 240
ECH23           CTX-S ADILRFTLLRSYRRDEWTAWMQAAGEHPPSPTHRVMVFDSSVTMLEAAQAGVGIAIAPVD 240
ATCC13047             ADILKFTLLRSYRRDEWTAWMQAAGENPPSPTHRVMVFDSSVTMLEAAQTGTGIAIAPVD 240
                      ****:*********************:**********************:*.********

ECH1                  MFTHLLASERIVQPFATQIELGSYWLTRLQSRAETPAMREFSRWLVEKMKK 291
ECH2                  MFTHLLASERIVQPFATQIELGSYWLTRLQSRAETPAMREFSRWLVEKMKK 291
ECH3                  MFTHLLASERIVQPFATQIELGSYWLTRLQSRAETPAMREFSRWLVEKMKK 291
ECH6                  MFTHLLASERIVQPFATQIELGSYWLTRLQSRAETPAMREFSRWLVEKMKK 291
ECH11                 MFTHLLASERIVQPFATQIELGSYWLTRLQSRAETPAMREFSRWLVEKMKK 291
ECH24                 MFTHLLASERIVQPFATQIELGSYWLTRLQSRAETPAMREFSRWLVEKMKK 291
ECH23           CTX-S MFTHLLASERIVQPFATQIELGSYWLTRLQSRAETPAMREFSRWLVEKMKK 291
ATCC13047             MFTHLLNSERIVQPFTTQIDLGSYWLTRLQSRAQTPAMREFAQWLVGKMQK 291
                      ****** ********:***:*************:*******::*** **:*
```

AmpD alignement

```
ATCC13047   CTX-S  ----MLLEDGWLVDARRVPSPHHDGRPEDEKPTLLVVHNISLPPGEFGGPWIDALFTGTI  56
ECH23       CTX-S  ----MLLENGWLVDARHVPSPHHDCRPEDEKPTLLVVHNISLPPGEFGGPWIDALFTGTI  56
ECH24              ----MLLENGWLVDARHVPSPHYDCRPEDEKPTLLVVHNISLPPGEFGGPWIDALFTGTI  56
ECH1               MSSSMLLENGWLVDARHVPSPHHDCRPEDEKPTLLVVHNISLPPGEFGGPWIDALFTGTI  60
ECH2               MSSSMLLENGWLVDARHVPSPHHDCRPEDEKPTLLVVHNISLPPGEFGGPWIDALFTGTI  60
ECH3               MSSSMLLENGWLVDARHVPSPHHDCRPEDEKPTLLVVHNISLPPGEFGGPWIDALFTGTI  60
ECH6               MSSSMLLENGWLVDARHVPSPHHDCRPEDEKPTLLVVHNISLPPGEFGGPWIDALFTGTI  60
ECH11              MSSSMLLENGWLVDARHVPSPHHDCRPEDEKPTLLVVHNISLPPGEFGGPWIDALFTGTI  60
                       ****:*******:*****:* ***********************************

ATCC13047   CTX-S  DPDAHPFFAEIAHLRVSVHCLIRRDGEVVQYVPFDKRAWHAGVSMYQGRERCNDFSIGIE  116
ECH23       CTX-S  DPDVHPFFAEIAHLRVSAHCLIRRDGEVVQYVPFDKRAWHAGVSMYQGRERCNDFSIGIE  116
ECH24              DPDVHPFFAEIAHLRVSAHCLIRRDGEVVQYVPFDKRAWHAGVSMYQGRERCNDFSIGIE  116
ECH1               DPDVHPFFAEIAHLRVSAHCLIRRDGEVVQYVPFDKRAWHAGVSMYQGRERCNDFSIGIE  120
ECH2               DPDVHPFFAEIAHLRVSAHCLIRRDGEVVQYVPFDKRAWHAGVSMYQGRERCNDFSIGIE  120
ECH3               DPDVHPFFAEIAHLRVSAHCLIRRDGEVVQYVPFDKRAWHAGVSMYQGRERCNDFSIGIE  120
ECH6               DPDVHPFFAEIAHLRVSAHCLIRRDGEVVQYVPFDKRAWHAGVSMYQGRERCNDFSIGIE  120
ECH11              DPDVHPFFAEIAHLRVSAHCLIRRDGEVVQYVPFDKRAWHAGVSMYQGRERCNDFSIGIE  120
                   ***.*************.******************************************

ATCC13047   CTX-S  LEGTDTTPYTDAQYQRLADITQTLIRLYPAIAENMTGHCDIAPARKTDPGPAFDWPRFRA  176
ECH23       CTX-S  LEGTDTTPYTDAQYEKLVAVTQTLIGRYPAIADNITGHSDIAPERKTDPGPAFDWSRFHA  176
ECH24              LEGTDTTPYTDAQYEKLVAVTQTLIGRYPAIADNITGHSDIAPERKTDPGPAFDWSRFHA  176
ECH1               LEGTDTTPYTDAQYEKLVAVTQTLIGRYPAIADNITGHSDIAPERKTDPGPAFDWSRFHA  180
ECH2               LEGTDTTPYTDAQYEKLVAVTQTLIGRYPAIADNITGHSDIAPERKTDPGPAFDWSRFHA  180
ECH3               LEGTDTTPYTDAQYEKLVAVTQTLIGRYPAIADNITGHSDIAPERKTDPGPAFDWSRFHA  180
ECH6               LEGTDTTPYTDAQYEKLVAVTQTLIGRYPAIADNITGHSDIAPERKTDPGPAFDWSRFHA  180
ECH11              LEGTDTTPYTDAQYEKLVAVTQTLIGRYPAIADNITGHSDIAPERKTDPGPAFDWSRFHA  180
                   **************::*. :*****  *****:*:***.**** *********** **:*

ATCC13047   CTX-S  MLTASSE----  183
ECH23       CTX-S  MLTTSSDKEIT  187
ECH24              MLTTSSDKEIT  187
ECH1               MLTTSSDKEIT  191
ECH2               MLTTSSDKEIT  191
ECH3               MLTTSSDKEIT  191
ECH6               MLTTSSDKEIT  191
ECH11              MLTTSSDKEIT  191
                   ***:**:
```
